# Impact of Nebulization on the Physicochemical Properties of Polymer–Lipid Hybrid Nanoparticles for Pulmonary Drug Delivery

**DOI:** 10.3390/ijms25095028

**Published:** 2024-05-05

**Authors:** Andrea Gonsalves, Jyothi U. Menon

**Affiliations:** 1 Department of Biomedical and Pharmaceutical Sciences, College of Pharmacy, University of Rhode Island, Kingston, RI 02881, USA; agonsalves@uri.edu; 2 Department of Chemical Engineering, University of Rhode Island, Kingston, RI 02881, USA

**Keywords:** Aeroneb, pulmonary drug delivery, nebulizer, vibrating-mesh nebulizer, aerosol, cancer, biomimetic, lung surfactant, macrophage

## Abstract

Nanoparticles (NPs) have shown significant potential for pulmonary administration of therapeutics for the treatment of chronic lung diseases in a localized and sustained manner. Nebulization is a suitable method of NP delivery, particularly in patients whose ability to breathe is impaired due to lung diseases. However, there are limited studies evaluating the physicochemical properties of NPs after they are passed through a nebulizer. High shear stress generated during nebulization could potentially affect the surface properties of NPs, resulting in the loss of encapsulated drugs and alteration in the release kinetics. Herein, we thoroughly examined the physicochemical properties as well as the therapeutic effectiveness of Infasurf lung surfactant (IFS)-coated PLGA NPs previously developed by us after passing through a commercial Aeroneb^®^ vibrating-mesh nebulizer. Nebulization did not alter the size, surface charge, IFS coating and bi-phasic release pattern exhibited by the NPs. However, there was a temporary reduction in the initial release of encapsulated therapeutics in the nebulized compared to non-nebulized NPs. This underscores the importance of evaluating the drug release kinetics of NPs using the inhalation method of choice to ensure suitability for the intended medical application. The cellular uptake studies demonstrated that both nebulized and non-nebulized NPs were less readily taken up by alveolar macrophages compared to lung cancer cells, confirming the IFS coating retention. Overall, nebulization did not significantly compromise the physicochemical properties as well as therapeutic efficacy of the prepared nanotherapeutics.

## 1. Introduction

Pulmonary drug delivery has emerged as a patient-compliant and non-invasive method of providing localized and systemic therapies to treat both pulmonary and non-pulmonary diseases [1]. This method of delivering therapies is suitable for treating various pulmonary diseases, such as chronic obstructive pulmonary disorder (COPD) and asthma, as it offers quick onset of action and minimal systemic side-effects as therapeutics are delivered directly to the lungs [2]. The lungs possesses a large surface area exceeding 100 m^2^ with an extremely thin alveolar epithelium ranging from 0.2 to 1 µm in thickness, which favors solute exchange and allows faster drug absorption and rapid drug onset following pulmonary administration [3,4]. Moreover, the relatively lower concentrations of drug-metabolizing enzymes in the lungs enhances drug bioavailability [5,6], while the non-invasiveness of the pulmonary route of delivery enhances patient compliance [7]. 

Therapeutics, upon inhalation, are deposited in the mucosal lining, from where they are subsequently carried via mucociliary advection and molecular diffusion to the lungs [8]. However, drugs administered via inhalation as aerosols or fine dry powders are often associated with poor retention properties and a shorter half-life, necessitating frequent dosing which may lead to increased local toxicity [9,10,11]. Additionally, most drugs exhibit limited water solubility and, therefore, cannot be aerosolized [4,12,13]. To overcome these limitations, nanotechnology-based drug delivery platforms are being increasingly explored [13,14,15]. In these platforms, drugs can be encapsulated within polymer or lipid nanocarriers, which are comparatively easier to disperse in aqueous solutions for aerosolization. Polymeric nanoparticles (NPs), in particular, facilitate controlled and sustained release of therapeutics via diffusion and polymer degradation, resulting in prolonged pharmacological effects at the desired site of action [9,16]. Additionally, polymers can readily be subjected to surface modifications and/or co-polymerized to augment the selectivity of polymeric NPs to further improve their therapeutic efficacy [15]. Beck-Broichsitter et al. successfully encapsulated sildenafil within a biodegradable and biocompatible polymer like poly(D,L-lactide-co-glycolide) (PLGA), showcasing a controlled and sustained release of the therapeutics in the treatment of pulmonary hypertension [16]. Similarly, Xu et al. developed methoxy poly(ethylene glycol)-poly(ethylenimine)-poly(l-glutamate) (mPEG-OEI-PLG) nanoparticles for co-delivery of doxorubicin and cisplatinum via pulmonary administration for lung cancer therapy [17].

Among the different approaches used for aerosol drug delivery, nebulization is an attractive option as it enables direct delivery of the inhaled nanotherapeutics in the form of fine droplets deep into the alveoli [18,19]. Nebulizers’ low inspiratory flow and normal ventilatory pattern are especially advantageous for delivering therapeutics to patients with lung cancer who experience difficulty with breathing. Upon aerosolization, nanoparticles with an average diameter below 1 micron form droplets with a mass median aerodynamic diameter ranging from 1 to 5 µm; this range is optimal for deposition of inhaled NPs deep in the alveolar region [20,21]. Gravitational settling, aerosol diffusion and inertial impaction are other factors that govern deposition, along with the physiochemical properties of nanoformulations [8]. We have previously reported that our PLGA NPs (average diameter: 160 nm) formed aerosol droplets in the 4–6 µm range upon nebulization, which would be appropriate for deposition in the distal lung consisting of the terminal bronchioles and alveoli [15].

However, materials arriving at the luminal surface of the alveoli tend to be actively eliminated by alveolar macrophages present in this region [22,23]. Multiple attempts have been made to alter the surface of polymeric NPs to prevent phagocytosis by alveolar macrophages so that they can be retained in the alveoli for longer periods of time for sustained therapy [24,25]. Pulmonary surfactants secreted by Type II alveolar cells in the lungs comprise a mixture of 80% of phospholipids, 10% of neutral lipids and 10% of surfactant proteins [26,27]. The phospholipids present in pulmonary surfactants have been found to form a protective barrier, thereby minimizing the internalization of nanoparticles by alveolar macrophages [28]. ONY Biotech’s Infasurf^®^ is a calf lung-derived lung surfactant that mimics the native lung surfactant produced by conducting the airways of the human lungs, and was therefore chosen for this research to shield NPs from clearance by macrophages [29]. We have recently reported the development of a biomimetic nanoplatform consisting of Infasurf^®^ lung surfactant-coated PLGA NPs, which was successful in evading alveolar macrophage uptake of NPs in vitro and improved NP retention following inhalation in vivo in mice [30]. Although single polymer/lipid NPs and hybrid NPs have been synthesized and characterized for delivering drugs to the lungs, there are limited studies that investigated the impact of nebulization on the physical and chemical attributes of these formulations. Detailed investigation on the effects of nebulization is necessary to ensure that the high shear stress developed during the nebulization process does not cause shear degradation of NPs [31], thereby resulting in the potential loss of coatings and encapsulated therapeutics, and changes in release behavior. To our knowledge, no studies have been performed to evaluate and compare the physicochemical, stability and in vitro effects of hybrid NP formulations consisting of multiple layers of lipids or polymers pre- and post-nebulization. The present work focuses on evaluating our Infasurf^®^ lung surfactant (IFS, ONY biotech, Amherst, NY, USA)-coated PLGA NPs (IFS_PLGA NPs) after passing through a commercial Aeroneb^®^ (Kent Scientific Corporation, Torrington, CT, USA) vibrating-mesh nebulizer. The PLGA core of these NPs contains paclitaxel (PTX), a potent anti-cancer drug which is used as a first-line treatment in lung cancer. PTX binds to microtubules in cells and induces cell cycle arrest, leading to apoptosis [32]. Due to its poor water solubility, PTX has been encapsulated within drug delivery vehicles such as micelles and albumin nanoparticles for therapy. Genexol-PM, Nanoxel and Abraxane are examples of commercially available PTX-based nanoformulations that are delivered intravenously [33]. The IFS_PLGA NPs described herein offer an alternative method of delivering therapies directly to the deep lung tissue by nebulization. The encapsulation of PTX within these NPs for anti-cancer drug delivery was explored herein. However, these NPs can also be used to deliver other anti-cancer agents for the treatment of lung cancer.

## 2. Results and Discussion

### 2.1. Fabrication and Evaluation of Nebulized NPs

**Figure 1:**
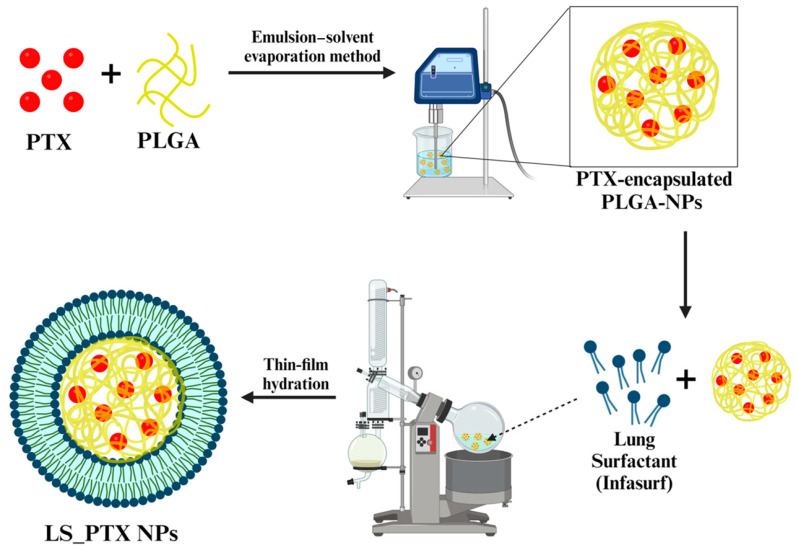
Schematic diagram illustrating the preparation of IFS_PLGA NPs. PTX is encapsulated within the polymeric matrix, followed by coating of the formulation by the IFS layer.

The IFS_PLGA NPs were successfully prepared by employing a two-step approach, wherein the PLGA NP core was first prepared by solvent evaporation, following which a thin film of lung surfactant was adsorbed on the surface of the PLGA NP core via hydration and sonication to form the final formulation (Figure 1). This adsorption is facilitated by electrostatic interactions between the positive charges of the zwitterionic dipalmitoylphosphatidylcholine (DPPC), a major component of the lung surfactant, and the negative surface charge of the polymeric core [34,35].

**Figure 2:**
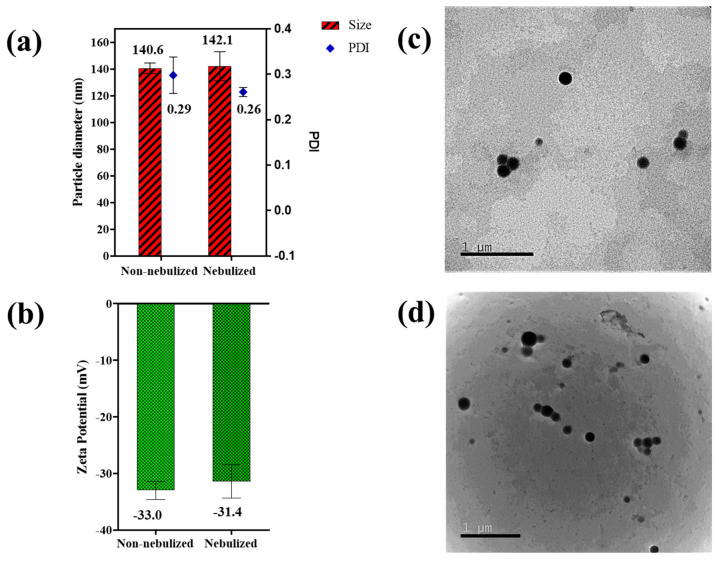
Physicochemical characterization of IFS_PLGA NPs pre- and post-nebulization: (**a**) NP size (nm) and polydispersity index (PDI), and (**b**) Zeta potential (mV) of nebulized and non-nebulized IFS_PLGA NPs (n = 3). TEM images of IFS_PLGA NPs: (**c**) pre-nebulization and (**d**) post-nebulization (scale: 1 µm).

During nebulization, the size of particles plays a critical role in influencing the mass median aerodynamic diameter. A greater number of nanoparticles can be incorporated within an aerosolized droplet as their size decreases, thus augmenting the deposition of therapeutics deep into the alveoli [36]. The prepared IFS_PLGA NPs were nebulized using an Aeroneb^®^ nebulizer. A dynamic light scattering (DLS) instrument was employed to evaluate the particle size, polydispersity index (PDI) and surface charge of the nebulized NPs, and these values were compared to the values obtained pre-nebulization. The DLS measurements, as shown in Figure 2a, demonstrated a minimal increase in particle size from 140.6 ± 3.87 nm to 142.1 ± 10.81 nm with a relatively stable PDI of 0.29 ± 0.04 and 0.26 ± 0.01 for the NPs before and after nebulization, respectively. Makled et al. developed a sildenafil citrate-containing solid lipid nanoparticle for treating pulmonary hypertension, and they observed similar results wherein the particles retained their sizes after nebulization, confirming the maintenance of colloidal stability after passing through a nebulizer [37]. Additionally, the zeta potential of the NPs pre- and post-nebulization displayed an insignificant overall change of −33.0 ± 1.60 mV and −31.4 ± 2.95 mV, respectively (Figure 2b). The uncoated PLGA NPs had a zeta potential of about −37.8 mV. The small, insignificant decrease in zeta potential noted after IFS coating was retained post nebulization. Since the zeta potential values of the NPs were greater than −30 mV, these particles were stable. Negatively charged particles have been found to accumulate more effectively within the pulmonary region compared to positively charged particles [38]; therefore, the negligible variations in zeta potential is favorable for deep lung deposition. Similar results were observed by Graczyk et al. in their study, wherein they reported no change in particle size (56 ± 10 nm) and zeta potential (~−44 mV) of a nebulized Rienso suspension (superparamagnetic iron oxide nanoparticles) [39]. The NP morphologies were also visualized using Transmission Electron Microscopy (TEM). For the nebulized samples, the nanosuspension was directly nebulized on the TEM grids before imaging. Nebulization did not impact the surface morphologies and size of the NPs, as shown in Figure 2c,d. The TEM images exhibit smaller particle diameters in comparison to the DLS measurements; this was expected as NPs are subjected to air-drying during TEM sample preparation, while DLS involves measuring the hydrodynamic diameter of particles in suspension.

### 2.2. Confirmation of the IFS Layer on the Nebulized IFS_PLGA NPs

The IFS-PLGA NP dispersion was passed through the nebulizer, and the nebulized fraction was evaluated for the presence of IFS coating via estimation of phospholipids using Stewart’s assay, a colorimetric assay that forms a colored complex between ammonium ferrothiocyanate and phospholipids, which can then be quantitatively measured [40]. As described above, Infasurf (IFS layer) is a calf-derived lung surfactant that comprises phospholipids (90%) and proteins (10%) [41,42]. The quantified phospholipids in the nebulized fraction were then compared to the non-nebulized formulation. As shown in Table 1, negligible loss of lipids was observed, confirming the retention of IFS coating on the NPs after passing through the nebulizer.

**Figure 3:**
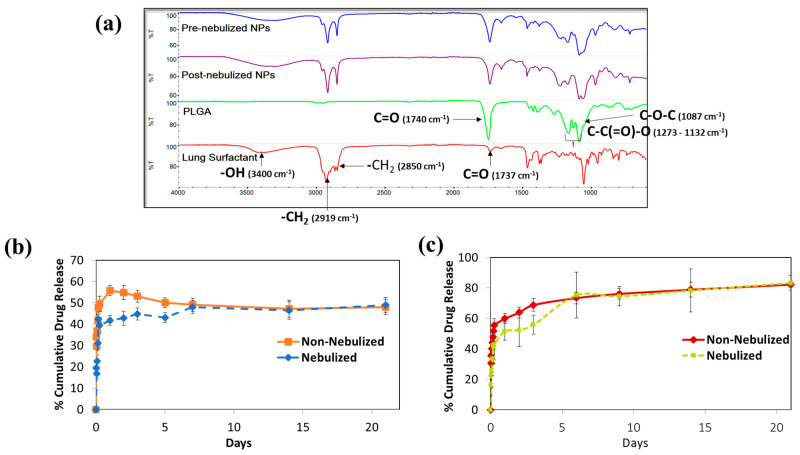
FTIR and drug release analysis of IFS_PLGA NPs pre- and post-nebulization. (**a**) FTIR spectra of IFS, PLGA and pre- and post-nebulized IFS_PLGA NPs, confirming the retention of all components after nebulization. (**b**) The cumulative PTX release profile from encapsulated IFS_PTX NPs pre- and post-nebulization at 37 °C. (**c**) The effect of nebulization on the release kinetics of the NP system using rhodamine-B as a model drug.

Additionally, FTIR spectra were obtained to study the impact of nebulization on the retention of the lipid coating on the polymeric PLGA NPs (Figure 3a). The FTIR spectra of both nebulized and non-nebulized IFS_PLGA NPs exhibited the characteristic peaks of IFS, showing the presence of -PO_2_ antisymmetric stretching double bonds at around 1223 cm^−1^, -C=O stretching observed at around 1737 cm^−1^, –CH_2_ symmetric stretching observed at 2850 cm^−1^, –CH_2_ antisymmetric stretching at 2919 cm^−1^, –CH_3_ stretching between 2955 and 2957 cm^−1^, and –OH group at 3400 cm^−1^ [43,44,45]. Additional peaks characteristic to PLGA at 1740 cm^−1^, representing the carbonyl stretching of lactide and glycolide; at 1221 and 1171 cm^−1^, attributed to the symmetric and asymmetric stretching of C–C(=O)–O; and at 1087 cm^−1^ due to the C-O-C stretching were also observed [46,47,48]. The FTIR findings affirmed that the nebulized IFS_PLGA NPs maintained their structural integrity and retained the IFS coating even after passing through the nebulizer.

### 2.3. Evaluation of Drug Loading in IFS-Coated NPs and In Vitro Release Behavior

PTX was loaded within the polymeric matrix, followed by IFS coating, as described in detail in Section 3.2. The drug-loaded NPs (IFS_PTX NPs) were evaluated for their drug loading capacity and encapsulation efficiency (EE%), which were estimated to be 73.7 ± 5.1% and 2.6 ± 0.98%, respectively, using a previously developed HPLC method [30].

Further investigation was conducted to evaluate the effect of nebulization using the Aeroneb^®^ vibrating-mesh nebulizer on the release kinetics of the IFS-coated polymeric NP system. Several studies have shown that the type of nebulizer used has varying effects on NP systems due to the technology used in the generation of aerosols [49,50,51]. Jet nebulizers utilize compressed air to generate aerosols and have been found to be have the most damaging effects on nanoformulations, particularly liposomes [52,53], while ultrasonic nebulizers are disadvantageous for thermo-sensitive formulations due to the generation of heat from piezoelectric crystals that are employed during aerosol generation [54]. Nebulizers utilizing the vibrating-mesh technology, on the other hand, demonstrated liposomal stability in the retention of drugs within liposomes [49]. As far as we know, there are no studies investigating the impact of nebulization on the release kinetics of nanoformulations. Regarding the present formulation with a two-layer system consisting of an IFS shell and a PLGA core, we hypothesized that the shear stress developed during nebulization might affect the NPs’ surface properties and, therefore, the release profile. As shown in Figure 3b, the nebulized IFS_PTX NPs had a similar drug release profile as the non-nebulized IFS_PTX NPs. The release kinetics of the IFS_PTX NPs pre- and post-nebulization in PBS at 37 °C demonstrated an initial burst release of 34 ± 10.45% and 19 ± 4.29% within the first hour, which is attributed to the bi-phasic release profile of the polymer. About 56 ± 2.35% and 42 ± 1.04% of the drug was released from the IFS_PTX NPs pre- and post-nebulization within 24 h, after which a cumulative release of about 45–49% of the encapsulated drug was observed for 21 days. However, the nebulized NPs displayed a decrease in the amount of drug released during the burst release phase; this decrease was observed until day 3, after which both groups maintained a similar drug release profile until day 21. The lower release from the nebulized NPs could be attributed to possible changes in membrane integrity and properties due to the shearing provided by the nebulizer, which could have impacted drug release. An overall decline in the release profile was observed in both groups of NPs, which is attributed to the base-catalyzed epimerization and hydrolysis of PTX in an aqueous solution reported by Sadegh et al. and which was also observed by us previously [30,55]. PTX contains ester groups that are hydrolytically sensitive. In water, the hydroxyl group in PTX undergoes initial epimerization, followed by base-catalyzed hydrolysis of PTX’s side chains and remaining ester bonds over time, which leads to the decreased cumulative release observed by us [56].

Due to the challenges in measuring PTX release from NPs over time, we used the chemically stable rhodamine-B as a model drug within the IFS_PLGA NPs to assess the drug release profiles of nebulized and non-nebulized NPs. The EE% of rhodamine-B-loaded IFS_PLGA NPs was determined to be 45.46 ± 2.10%. As shown in Figure 3c, a similar overall trend in the release profile was observed for the rhodamine-B-loaded IFS_PLGA NPs pre- and post-nebulization. Both groups demonstrated an initial burst release of 30.74 ± 0.75% and 16.17 ± 6.26% of the encapsulated drug in 30 min for the non-nebulized and nebulized NPs, respectively. This was followed by a sustained release of about 82.32 ± 1.88% and 83.35 ± 4.99% of the encapsulated drug from the non-nebulized and nebulized NPs, respectively, within 21 days.

### 2.4. Nebulization Performance of IFS_PTX NPs Using Aeroneb^®^

**Figure 4:**
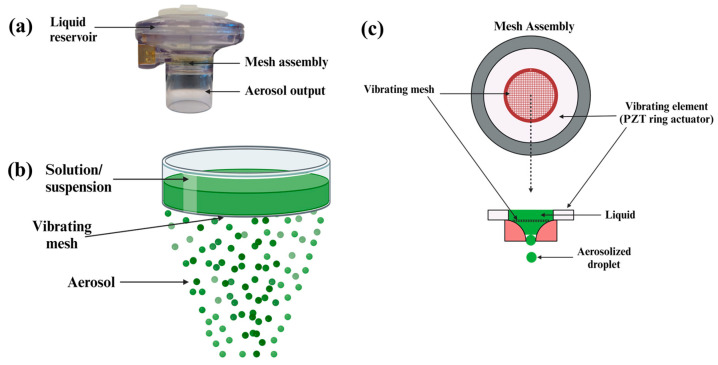
(**a**) Commercially available Aeroneb^®^ nebulizer. (**b**) Schematic representation of the vibrating-mesh technology. (**c**) Schematic representation of the mesh assembly within the nebulizer [60,62].

Parameters associated with the type of nebulizer used can affect nebulization performance, which, in turn, impacts the nebulization efficiency and therapeutic efficacy [57,58]. The Aeroneb^®^ nebulizer works on the principle of vibrating-mesh technology, wherein liquid droplets pass through the mesh that is vibrating due to the piezo-element to form aerosolized droplets [59,60,61]. IFS_PTX NPs were passed through the nebulizer, and the nebulization efficiency, nebulization rate and aerosol output rate were evaluated (Figure 4). The nebulizer was able to successfully nebulize 2 mL of the NP suspension (1 mg/mL) in about 7 ± 0.95 min, displaying a nebulization efficiency of 89.03 ± 3.0%. An aerosol output rate of 261.9 ± 45.1 mg/ min was determined, while approximately 7.5 ± 3.7% of the NP suspension was estimated to have been lost within the inner walls of the liquid reservoir and the aerosol output (Table 2).

When compared to other nebulizers such as ultrasonic and jet nebulizers, a vibrating-mesh nebulizer is potentially advantageous for several reasons, namely less noise, excellent delivery efficiency, low residual dose and minimal change in the drug concentration during nebulization as there is no temperature change, making it favorable for nebulizing temperature-sensitive therapeutics [63]. The nebulized IFS_PTX NPs were evaluated for any change in the drug concentration. No change in the drug concentration was observed, confirming our formulation was stable enough to withstand the vibrational energy and any drug loss was prevented during the nebulization process.

### 2.5. In Vitro Studies of Nebulized NPs

**Figure 5:**
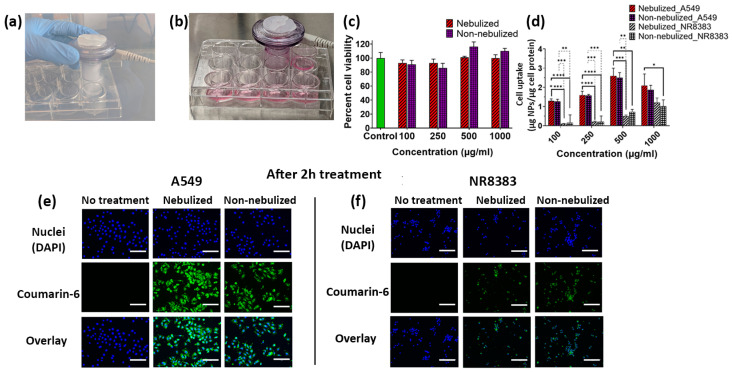
Photographs demonstrating (**a**) the use of a nebulizer directly on the cells and (**b**) condensation of the nebulized fraction on the cells. (**c**) MTT assays carried out on A549 cells to investigate the effect of the nebulizer on cytocompatibility. (**d**) Cellular uptake results depicting the uptake kinetics of nebulized NPs by A549 and NR8383 cells. p-value: * = *p* ˂ 0.05, ** = *p* ˂ 0.01, *** = *p* ˂ 0.001, and **** = *p* ˂ 0.0001, showing a significant difference between the two groups. EVOS images demonstrating cellular uptake of coumarin-6-labeled IFS_PLGA NPs at an NP concentration of 500 µg/mL by the (**e**) A549 and (**f**) NR8383 cell lines (green = coumarin-6, blue = DAPI, scale bar = 200 µm, magnification = 20×).

The effect of nebulized blank IFS_PLGA NPs was then studied on A549 lung adenocarcinoma cells using MTT assays. The cells were directly nebulized, as shown in Figure 5a,b, with an NP dispersion ranging from 100 to 1000 µg/mL. As shown in Figure 5c, greater than 85% cell viability was exhibited by the cells treated with IFS_PLGA NPs for up to a concentration of 1000 µg/mL, indicating that the NPs were cytocompatible at high concentrations. For comparison, the cells were also treated with non-nebulized NPs (NP suspension directly added to the cells). There was no significant difference in cell viability between the tested groups.

Furthermore, a cellular uptake study was conducted to determine the nebulization effects on NP uptake by both A549 and NR8383 cells. For this investigation, coumarin-6 dye was encapsulated within the polymeric matrix of the NP system, as described in Section 3.2 and Section 3.10. Coumarin-6 is relatively stable within encapsulated systems, with no immediate leakage of the dye into cell culture media. Additionally, it does not produce any acute cell toxicity [64]. We had previously reported a statistically significant reduction in alveolar macrophage uptake of our developed IFS_PLGA NPs when compared to PLGA NPs not coated with IFS, indicating that the biomimetic IFS coating minimized recognition and subsequent phagocytosis by alveolar macrophages [30]. Figure 5d–f demonstrate the cellular uptake of nebulized and non-nebulized IFS_PLGA NPs by the A549 and NR8383 cell lines. As observed previously by our group, significant NP uptake by the A549 cells was observed when compared to the alveolar macrophages (NR8383) [30]. The nebulization process did not seem to significantly impact the uptake of NPs by either cell line. These findings further confirm the retention of the IFS coating on the PLGA NPs when passing through the vibrating-mesh nebulizer, which prevented NR8383 alveolar macrophage-mediated phagocytosis of these IFS_PLGA NPs.

### 2.6. Effect of Nebulization on In Vitro Therapeutic Efficacy 

**Figure 6:**
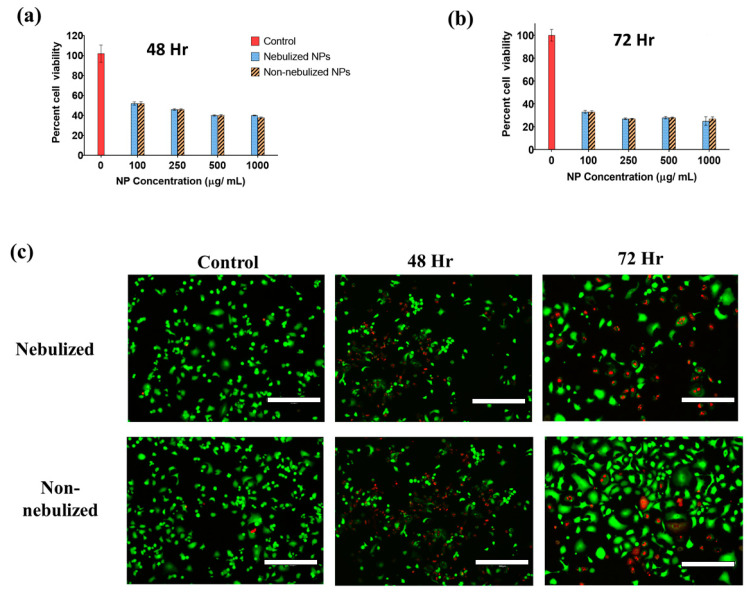
(**a**) In vitro studies investigating the therapeutic effectiveness of nebulized particles in comparison with non-nebulized NPs at 48 h and (**b**) 72 h. (n = 4, mean ± standard deviation). (**c**) Live/dead staining of A549 cells after treatment with nebulized and non-nebulized IFS_PTX NPs at 250 µg/mL (scale bar = 300 µm, magnification = 10×).

The therapeutic efficacy of nebulized IFS_PTX NPs was subsequently investigated against the A549 cell line. The cells were dosed either by direct nebulization or by pipetting varying concentrations (100–1000 µg/mL) of IFS_PTX NPs, as described in detail in Section 3.11. Following 2 h of incubation with the NPs, the cells were washed, and the medium was replaced. The washing step was performed so that the results obtained would be solely due to the release of the drug from the NPs taken up within the first two hours of treatment. As previously observed by our group, a dose-dependent cytotoxic effect (Figure 6a–c) was noted when the cells were treated with drug-loaded NPs for 2 h [30]. The method of NP administration, however, had no impact on the cytotoxic effect, further confirming that the drug release kinetics and therapeutic effects of the LS-coated NPs were not impacted upon nebulization. Comparable results were also observed by Verma et al. in their study, wherein no significant changes in cell death was observed when A549 cells were treated with quercetin-loaded magnetic NPs administered via direct pipetting or nebulization [65]. Vencken et al. also demonstrated that nebulization of microRNA-17-loaded lipid–polymer hybrid nanoparticles was able to knockdown interleukin (IL)-8, further confirming that the nebulization process did not have any impact on the therapeutic efficacy of NPs [66].

## 3. Materials and Methods

### 3.1. Materials

PLGA [Poly(D,L-lactide-co-glycolide) or Resomer RG 503 H, at a copolymer ratio of 50:50], cholesterol, PVA [poly (vinyl alcohol), MW: 13,000–23,000], chloroform and PBS (phosphate-buffered saline: 0.01 M phosphate buffer, 0.0027 M potassium chloride and 0.137 M sodium chloride, pH 7.4) were purchased from Sigma–Aldrich (St. Louis, MO, USA). Infasurf (Calfactant) was a generous gift from Dr. Edmund Egan and ONY, Inc. (Amherst, NY, USA). PTX and acetonitrile were procured from Alfa Alesar (Ward Hill, MA, USA). Analytical-grade chemicals and reagents were used in this research.

NR8383 alveolar macrophage (CRL-2192) and A549 lung adenocarcinoma cells (CCL-185^TM^) were purchased from ATCC (Manassas, VA, USA). DMEM [Dulbecco’s minimum essential medium] was purchased from Sigma–Aldrich (St. Louis, MO, USA). F12-K culture medium, 0.25% trypsin, FBS [fetal bovine serum] and pen-strep [penicillin–streptomycin antibiotic] were purchased from Gibco (Grand Island, NY, USA). The bicinchoninic acid (BCA)^®^ Protein Assay Kit was obtained from Pierce, Rockford, IL, USA.

### 3.2. NP Synthesis

The PTX-encapsulated PLGA NPs were prepared via an emulsion–solvent evaporation technique. In brief, PTX (5 mg) and PLGA (50 mg) were dissolved in 5 mL of chloroform, which was then added dropwise into 5% *w*/*v* PVA solution under constant stirring and emulsified with the help of a probe sonicator (Fisher Scientific CL-18, Waltham, MA, USA) for 3 min. To ensure complete removal of the organic solvent, the resultant emulsion was stirred overnight. The NPs were purified via ultracentrifugation (Optima L-100 XP, Beckman Coulter, Brea, CA, USA) at 25,000 g at 10 °C for 30 min. The NPs were then lyophilized and stored at −20 °C for future use.

Further, the prepared NPs were coated with IFS via thin-film hydration technique. IFS (5 mg) and cholesterol (molar ratio of lipid to cholesterol = 15:1) dissolved in 2 mL chloroform was dried into a thin film under reduced pressure at 50 °C by employing a rotary evaporator (R-100, Buchi, New Castle, DE USA). The lipid film obtained was then hydrated at 50 °C for 1 h with 4 mL of 0.5 mg/mL PTX-encapsulated PLGA NP dispersion, followed by probe sonication for 3 min. The prepared IFS-coated PTX-encapsulated PLGA NPs (IFS_PTX NPs) were then purified for 24 h using a dialysis bag (12–14 kDa molecular weight cutoff) that was preequilibrated. The dialysate medium (Milli-Q water) was replaced every 3–4 h. The purified NPs were then freeze-dried and stored at −20 °C until future use.

### 3.3. Characterization of Particle Size and Morphology of the NPs

The NPs were evaluated for their physicochemical characteristics pre- and post-nebulization. The NP size, polydispersity index (PDI) and zeta potential (ZP) were measured by a dynamic light scattering analyzer (DLS) (NanoZS, Malvern, PA, USA). The shape and morphology of the IFS-coated NPs pre- and post-nebulization were evaluated by a JEM-F200 transmission electron microscope (TEM) (JEOL, Peabody, MA, USA). In short, the NP suspension droplets were mounted on carbon-coated TEM grids and air-dried before visualization under the microscope. For the nebulized samples, NPs dispersed in MilliQ water (1 mg/mL) were passed through the Aeroneb nebulizer. The nebulized NPs were then analyzed for their size and morphology in the same manner as described above.

### 3.4. Confirmation and Quantification of the IFS Coating

Fourier-transform infrared spectroscopy (FTIR) (Jasco FT-IR Spectrophotometer, JASCO, Easton, MD, USA) was employed to study the retention of the IFS coating on the nebulized NPs. FTIR spectra were obtained at ambient temperature in the range from 400 to 4000 cm^−1^ to confirm the incorporation of the polymer, lipid and drug in the final formulation, and to study potential chemical interactions between them. The Omnic^TM^ FTIR Software was used to identify and interpret the different functional and vibrational peaks.

Furthermore, Stewart’s phospholipid colorimetric assay was used to confirm and quantify the IFS coating on the nebulized NPs [40,67]. Briefly, 1 mg/mL of blank NPs (IFS_PLGA-NPs) were passed through the nebulizer, collected, and then lyophilized. The lyophilized NPs were then dissolved in chloroform (2 mL) and vigorously vortexed for 1 min with ammonium ferrothiocyanate (2 mL) that was freshly prepared. The resultant mixture was then allowed to stand for 15 to 20 min to aid in the phase separation. The extracted phospholipid present in the lower chloroform layer was then measured using a GenesysTM 50 UV-Vis spectrophotometer (ThermoFisher Scientific, Waltham, MA, USA) at 470 nm.

### 3.5. Assessment of PTX Encapsulation Efficiency and Loading in IFS-Coated NPs

High-performance liquid chromatography (HPLC) (Schimadzu Corporation, Columbia, MD, USA) was used to quantify the PTX content in the NPs. A reverse-phase analysis was performed using an EC 125/4.6 Nucleosil 100-5 C 18 column (4.6 mm × 125 mm, pore size 5 μm, Macherey-Nagel, Allentown, PA, USA). The mobile phase consisted of a mixture of acetonitrile and water at a ratio of 50:50, with a flow rate of 1.0 mL/min. The column temperature was set at 30 °C and the injection volume was 20 μL. PTX detection was conducted at a wavelength of 227 nm.

Encapsulation efficiency (EE) and drug loading efficiency (LE) were determined by first breaking down the NPs using acetonitrile and then vortexing the suspension vigorously to ensure the complete release of the entrapped PTX, after which an equal volume of PBS was added. The resultant mixture was filtered and then injected into the HPLC. EE is the fraction of the drug used initially for encapsulation that was loaded into the NPs. LE is the amount of drug loaded per unit weight of the NPs. The EE and LE were calculated as shown below:

$$EE\left(\%\right)=\frac{Amount\;of\;drug\;in\;nanoparticles\;(mg)}{Initial\;amount\;of\;drug\;used\;(mg)}\times 100$$

$$LE\left(\%\right)=\frac{Amount\;of\;drug\;in\;nanoparticles\;(mg)}{Total\;amount\;of\;NP\;(mg)}\times 100$$

### 3.6. In Vitro Drug Release Studies

IFS_PTX NP (2 mg) dispersion in PBS (1 mL) was added to a dialysis bag (molecular weight cut-off (MWCO) of 12–14 kDa). The bag was immersed in screw-capped tubes containing 5 mL of PBS, which was then placed on an orbital shaker set to 75 rpm at 37 ± 0.5 °C for 21 days. At predefined timepoints, 0.5 mL of the dialysate was collected. This was replaced with 0.5 mL of pre-warmed PBS. The released PTX from the NPs was quantified using the HPLC method as detailed above.

The NPs were also evaluated for their release kinetics post nebulization. For this, 1 mg/mL NPs was first passed through the Aeroneb^®^ nebulizer (flow rate: >0.1 mL/min, volume mean diameter (VMD): 4.0–6.0 µm, residual volume: <0.2 mL), collected, and then lyophilized to determine the exact amount of particles used per sample. The amount of drug released from the nebulized NPs was assessed as detailed above, and non-nebulized NPs were used as a control. Since PTX tends to undergo epimerization and base-catalyzed hydrolysis when in aqueous media [55], rhodamine-B dye was used as a model drug for encapsulation within the PLGA NPs in these drug release studies. To synthesize rhodamine-B-containing NPs, the same preparation steps as described in Section 3.2 were followed, with the exception that PTX was replaced with 1 mg of rhodamine-B.

### 3.7. Evaluation of Nebulization Performance of IFS_PTX NPs

The Aeroneb^®^ nebulizer was evaluated for its nebulization performance following the passage of NPs through it. Briefly, 2 mL of a 1 mg/mL NP suspension was passed through the nebulizer, and the mass and volume of the collected fraction was assessed. The nebulizer was then investigated for the following parameters [37]:

$$Nebulization\;Efficiency,NE\left(\%\right)=\frac{Mass\;of\;the\;collected\;nebulized\;fraction\;\left(mg\right)}{Total\;mass\;of\;NPs\;instilled\;in\;the\;nebuliuzer\;\left(mg\right)}\times 100$$

(1) $$Fluid\;output\;rate\;\left(\frac{mg}{min}\right)=\frac{Mass\;of\;the\;collected\;nebulized\;fraction\;(mg)}{Time\;taken\;to\;nebulize\;the\;NPs\;(min)}\times 100$$

(2) $$Percentage\;remaining\;\left(\%\right)=\frac{Mass\;of\;fluid\;remaining\;in\;the\;nebulizer\;post\;nebulization\;(mg)}{Total\;mass\;of\;NPs\;instilled\;in\;the\;nebuliuzer\;(mg)}\times 100$$

Additionally, drug loss from the IFS_PTX NPs following nebulization was also evaluated. A total of 2 mL of 1 mg/mL of IFS_PTX NPs suspension was passed through the nebulizer, and the collected nebulized fraction was centrifuged. An equal volume of acetonitrile was added to the supernatant, and the resultant solution was then injected into the HPLC and quantified as described above in Section 3.5.

The drug loss of the nebulized NPs was calculated as follows:

(3) $$Drug\;Loss\left(\%\right)=\frac{Amount\;of\;drug\;present\;in\;nebulized\;fraction}{Amount\;of\;drug\;present\;in\;non-nebulized\;fraction}\times 100$$

### 3.8. Cell Culture Conditions

A549 lung adenocarcinoma cells were cultured in complete DMEM cell culture medium containing 10% *v*/*v* FBS and 1% *v*/*v* pen-strep solution. Complete Ham’s F-12 medium containing 15% *v*/*v* FBS and 1% *v*/*v* pen-strep solution was used for culturing NR8383 alveolar macrophage cells. The cells were cultured in a 5% CO_2_ incubator at 37 °C.

### 3.9. In Vitro Cytocompatibility Studies Using IFS_PLGA NPs

The cytocompatibility of the nebulized and non-nebulized NPs was evaluated using A549 cells by MTT colorimetric assays (Vybrant MTT Cell Proliferation Assay Kit, Thermo Fisher Scientific, Waltham, MA, USA). The cells were initially seeded at a seeding density of 10 × 10^3^ cells/well in a 12-well plate, followed by 24 h incubation at 37 °C to facilitate cell attachment. Post 24 h, the medium in the wells was removed and varying concentrations of IFS_PLGA NPs (0, 250, 500 and 1000 µg/mL) suspended in the medium were directly nebulized on the cells. The cells were then allowed to incubate for 24 h at 37 °C. For comparison, cells were also directly treated with non-nebulized NPs at similar NP concentrations. The untreated cells served as the control in this study. Following 24 h of treatment, the cells were gently washed using PBS, followed by the addition of the MTT reagent. Absorbance readings at 540 nm were obtained using a Synergy H1 microplate reader (BioTek, Winooski, VT, USA). Cell viability was calculated as the survival percentage of treated cells relative to the untreated control.

### 3.10. In Vitro Cellular Uptake of IFS_PLGA NPs

The effects of nebulization on the cellular internalization of the prepared NPs were also determined. For this investigation, IFS_PLGA NPs containing coumarin-6 dye were prepared as described in Section 3.2, except that PTX was replaced with coumarin-6 (1 mg). Briefly, the A549 and NR8383 cell lines were seeded at a seeding density of 10 × 10^3^ cells/well in a 12-well plate and allowed to attach at 37 °C for 24 h. Prior to the NP treatment, the cells were starved using a serum-free medium for 2 h. They were then treated with different NP concentrations ranging from 0 to 1000 µg/mL for 2 h. The two groups studied in this experiment were nebulized and non-nebulized NPs. In the nebulized NP group, the cells were directly exposed to the NPs that passed through the nebulizer, while in the non-nebulized group, the cells were directly treated with NPs suspended in the medium. Cells that were not treated with NPs served as a control. Following gentle washing in PBS at the end of the experiment, the cells were lysed using Triton X-100. NP uptake was determined by measuring NP fluorescence intensity, which was normalized to the total cell protein content quantified with the help of a Pierce BCA protein assay in each well. Cellular uptake was also qualitatively studied with the help of the EVOS^®^ FL Auto Imaging System (ThermoFisher Scientific, Waltham, MA, USA). For imaging NP uptake, cells seeded at 20 × 10^3^ cells/well in a 48-well plate were treated in a similar manner as mentioned above. Following the NP treatment for 2 h, these cells were washed gently with PBS, fixed using 4% formaldehyde, and imaged after staining the nuclei with DAPI.

### 3.11. In Vitro Therapeutic Efficacy Studies

The nebulized drug-loaded NPs (IFS_PTX NPs) were also investigated for anti-cancer effects with the help of an MTT assay. In this investigation, A549 cells seeded at a density of 10 × 10^3^ cells/well in a 12-well plate and incubated for 24 h were directly exposed to different drug-loaded NP concentrations (0, 125, 250 and 500 µg/mL) for 2 h. Post 2 h of incubation, the treatment medium was discarded, and the cells were washed with PBS to remove NPs that were not taken up. Then, the cells were incubated for 48 and 72 h in a drug-free medium. The MTT assay was performed to determine cell viability.

Additionally, the anti-proliferative activity of nebulized IFS_PTX NPs were also investigated using LIVE/DEAD staining. Briefly, A549 cells were seeded and treated with varying concentrations of IFS_PTX NPs for 2 h as described above. After 2 h, as described above, the treatment medium was removed, and the cells were washed with PBS. Fresh medium was added, and the cells were incubated for 48 and 72 h. After a specific time interval, the cells were gently washed twice with PBS and stained with LIVE/DEAD stain as instructed by the manufacturer. The stained cells were then imaged using the EVOS^®^ FL Auto Imaging System. Cells receiving no treatment served as the control group.

### 3.12. Statistical Analysis

All experiments were conducted independently in quadruplicate and the outcomes were presented as mean ± deviation. Statistical analysis was conducted using the GraphPad Prism software, with the significance levels denoted as * *p* < 0.05, ** *p* ˂ 0.01, *** *p* ˂ 0.001 and **** *p* ˂ 0.0001.

## 4. Conclusions

Pulmonary drug delivery is a convenient and patient-compliant method for delivering therapies in a localized manner to treat chronic pulmonary conditions, including lung cancer. Due to the weakened breathing capacity of patients with lung cancer, synchronization of inhalation with the inhaler device actuation is challenging. Nebulization of drug-loaded NPs is an attractive approach for localized delivery of medications for sustained therapy. In this study, we investigated in detail the physicochemical properties and therapeutic efficacy of our previously developed IFS-PLGA NPs upon passing through a commercial Aeroneb^®^ vibrating-mesh nebulizer. Nebulization did not impact the size, surface charge and retention of the IFS coating of the NPs, which was confirmed by DLS, FTIR and Stewart’s assay. However, a decrease in the initial release of the encapsulated therapeutics by the nebulized NPs was observed up to day 3 when compared to the non-nebulized NPs. A key contribution of this work to pulmonary drug delivery research is that it highlights the importance of testing the drug release kinetics of inhalable nanoformulations using a chosen mode of inhalation during preclinical investigations to ensure that the release is appropriate for the desired application. Nebulization of NPs through the vibrating-mesh nebulizer had no significant impact on therapeutic efficacy of the NPs. Cellular uptake studies confirmed that nebulized NPs were taken up less by alveolar macrophages than by lung cancer cells, confirming the retention of the IFS coating. Future work will involve in vivo evaluation of NPs post nebulization to investigate the biodistribution, safety and therapeutic effects of aerosolized NPs using established tumor models.

## Figures and Tables

**Table 1:** Estimation of phospholipids pre- and post-nebulization.

Mg of Lipids/Mg of IFS_PLGA NPs	
Before nebulization	After nebulization
0.53 ± 0.07	0.50 ± 0.03

**Table 2:** Nebulization performance of IFS_PTX NPs using Aeroneb^®^.

Nebulization efficiency (%)	89.03 ± 3.0
Time to nebulize (min)	7 ± 0.95
Fluid output rate (mg/min)	261.99 ± 45.09
Remaining percentage (%)	7.53 ± 3.65

## Data Availability

The data generated in this study are available upon request.
